# Seizures, deep vein thrombosis, and pulmonary emboli in a severe case of May–Thurner syndrome: a case report

**DOI:** 10.1186/s13256-022-03639-6

**Published:** 2022-11-11

**Authors:** Kevin D. Seely, Heidi J. Arreola, Loveleen K. Paul, Jordan A. Higgs, Benjamin Brooks, Randal C. Anderson

**Affiliations:** College of Osteopathic Medicine, Rocky Vista University, Ivins, UT 84738 USA

**Keywords:** May–Thurner syndrome, Left common iliac vein compression syndrome, Case report, Deep vein thrombosis, Ultrasound, Computer tomography venography, Interventional radiology, Stent

## Abstract

**Background:**

May–Thurner syndrome is a vascular disorder caused by the right common iliac artery compressing the left common iliac vein against the lumbar spine, causing distal venous stasis and potentially leading to fibrous change in the venous wall structure. Although May–Thurner syndrome is most commonly discovered in females upon investigation of new-onset deep vein thrombosis, we present the case of an otherwise healthy 29-year-old male with severe May–Thurner syndrome who presented with seizures, bilateral deep vein thrombosis, and diffuse pulmonary emboli. Seizures constituted the earliest presenting symptoms for the patient. Although it is difficult to prove that the patient’s seizures were related to the May–Thurner syndrome, this possible association renders this case extraordinary.

**Case presentation:**

This report describes the case of a 29-year-old previously healthy white male with a severe case of left-sided May–Thurner syndrome that required extensive medical and interventional treatment. The patient experienced two seizures, one month apart, both of which occurred while residing at high altitude. The patient had no prior history of seizures, and epilepsy was ruled out. Three weeks after the second seizure, he presented to the emergency room with hemoptysis, dyspnea, and severe leg pain. Sites of thrombus were confirmed in both legs and diffusely in the lungs. Etiological work-up after treatment with intravenous tissue plasminogen activator revealed May–Thurner syndrome. Hematology workup including genetic testing showed no evidence of coagulopathy. Bilateral common iliac venous stents were placed to attempt definitive treatment. Despite stenting, the patient had another thrombotic event with associated sequelae after discontinuation of anticoagulation. The patient has not had another seizure since the stents were placed. Despite the negative testing, the patient remains on lifelong chemoprophylaxis in the event of an undiscovered hypercoagulopathy.

**Conclusions:**

The care team theorizes that the seizures resulted from hypoxia due to May–Thurner syndrome-induced hemostasis and associated thrombotic events, the high-altitude location of his residence at the time he experienced the seizures, and shallow breathing during sleep. For patients with lower limb venous thrombosis, May–Thurner syndrome should be considered in the differential diagnosis. Endovascular treatment followed by extended prophylactic anticoagulation therapy until the patient is determined to be no longer at risk for thrombosis is recommended. Post-venoplasty thrombosis is a common complication of endovascular treatment of May–Thurner syndrome and should be carefully monitored.

## Background

May–Thurner syndrome (MTS) is an anatomical vascular disease caused by the right common iliac artery compressing the left common iliac vein against the lumbar spine, which may result in deep vein thrombosis (DVT) in the distal lower limb [1]. The disease is predominately left-sided, although variations are known to affect the right lower limb [2–4]. MTS is most commonly diagnosed in biological females, however, reported cases in young biological males have been increasing in number [1].

MTS is an uncommon cause of DVT, accounting for approximately 2–5% of lower-extremity venous disorders [5]. Risk factors for MTS include female sex, multiparity, use of oral contraceptives, scoliosis, dehydration, hypercoagulable disorders, and cumulative radiation exposure [6–14]. Few cases of MTS in young males have been reported in literature. Hung *et al.* [15] reported a case of an otherwise healthy 23-year-old male with no known risk factors who was found to have MTS after presenting with diffuse lower limb thrombosis upon work-up of leg pain which was treated without complication.

We present the case and associated imaging of an otherwise healthy 29-year-old male who presented first with generalized seizure and shortness of breath and was found to have bilateral pulmonary emboli and left lower limb DVT. MTS was discovered upon work-up of the differential diagnosis which included malignancy and coagulopathy (“Case presentation” section). His treatment course was complicated by post-venoplasty thrombosis with pulmonary embolism (PE), which was treated with pharmacomechanical thrombectomy. Finally, we discuss the diagnosis and various treatment approaches in “Discussion” section and conclude with valuable clinical takeaways for the diagnosis and management of patients with MTS (“Conclusion” section).

## Case presentation

A 29-year-old white male was transported to the emergency department by ambulance after experiencing a seizure in the early morning hours in September 2018. He had experienced mild pain in his left leg 1 day earlier, but did not experience any shortness of breath or chest pain at this time. Past medical history included minor mitral valve prolapse diagnosed in adolescence and at age 18 years a left tibial tubercle fracture. The patient was a commercial airline pilot and reported an average of 18 days or 90 hour per month spent piloting. He was living in a high-altitude city at the time of symptom presentation. Family history included a cousin with epilepsy and was otherwise non-contributory. The patient was discharged after an unremarkable head computed tomography (CT) scan and was referred to neurology. Neurology ruled out epilepsy as the cause of his seizures. Additional work-up, including electroencephalogram (EEG), was within normal limits.

One week after his first seizure and subsequent neurology consult, the patient presented at urgent care with left leg pain and exertional shortness of breath. No imaging or bloodwork was ordered at this time. He was discharged with an erroneous diagnosis of allergies. Later in the same month, he experienced his second seizure, again in the early morning hours, but did not seek emergent medical care. Upon a second evaluation by the neurologist, he was prescribed levetiracetam.

In November 2018, the patient presented to the emergency department with 3-day history of shortness of breath, hemoptysis, left calf pain radiating to the popliteal area, and intermittent left midsternal chest pain. The patient underwent an extended period of automobile travel of approximately 20 hour duration 2 weeks prior. The patient’s vitals were BP 145/88, MAP 107 mmHg, temperature 97.8 °F, HR 93, RR 16, and SaO_2_ of 95% on RA. Upon physical examination, lungs were clear to auscultation bilaterally with no diminished breath sounds. Cardiac auscultation revealed no gallops or murmurs. Radial and femoral pulses were normal bilaterally. No edema was found in the extremities. The neurological examination was within normal limits.

**Fig. 1:**
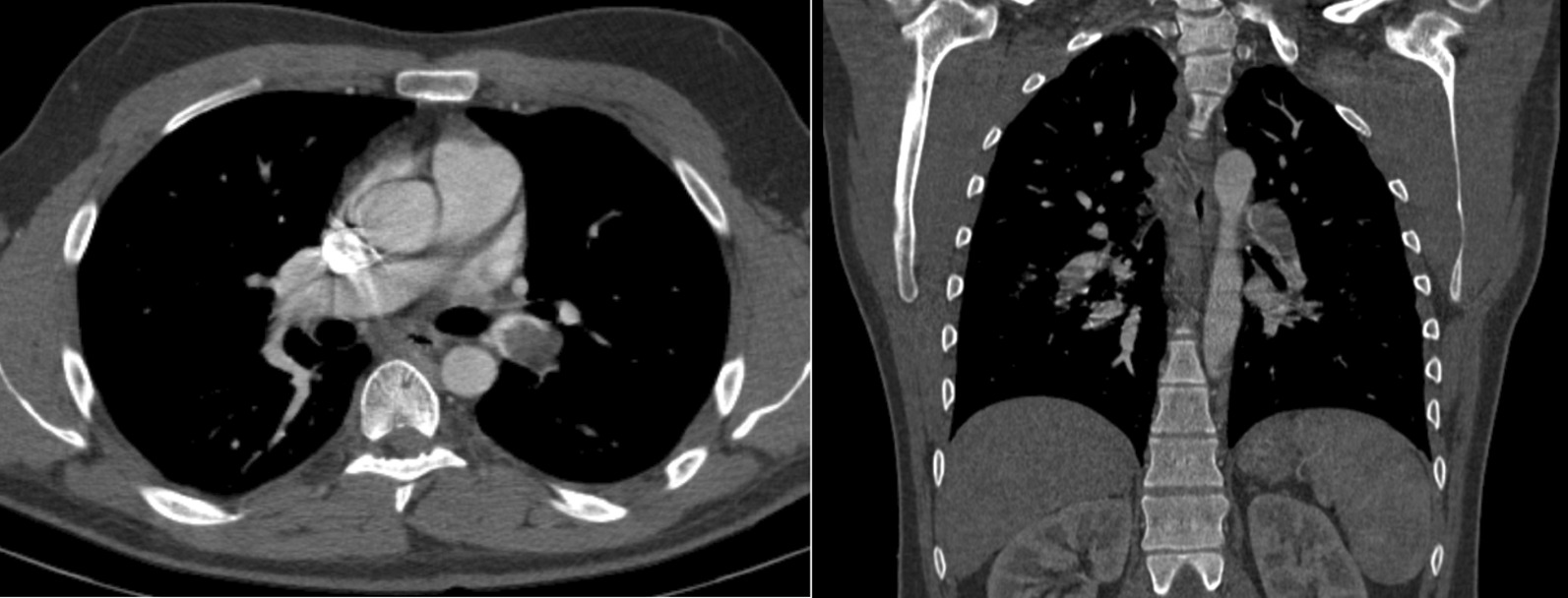
(Left) an axial slice from a CT of the chest obtained during the pulmonary angiographic phase of IV contrast administration demonstrates the largest site of PE in the patient, located in the left proximal interlobar pulmonary artery. (Right) a coronal view demonstrating PE located in the left proximal interlobar pulmonary artery

Blood work and imaging were subsequently ordered. Electrocardiogram (ECG) showed normal sinus rhythm and a rate of 98 bpm. D-dimer was elevated, which prompted further testing. Chest CT chest with intravenous (IV) contrast showed extensive bilateral pulmonary embolism (Fig. 1). Clot burden was identified in left and right interlobar arteries extending into the segmental and subsegmental branches without infarction or effusion. IV tPA was administered. A 4-mm lingular nodule was found on CT, and oncological work-up was recommended.

**Fig. 2:**
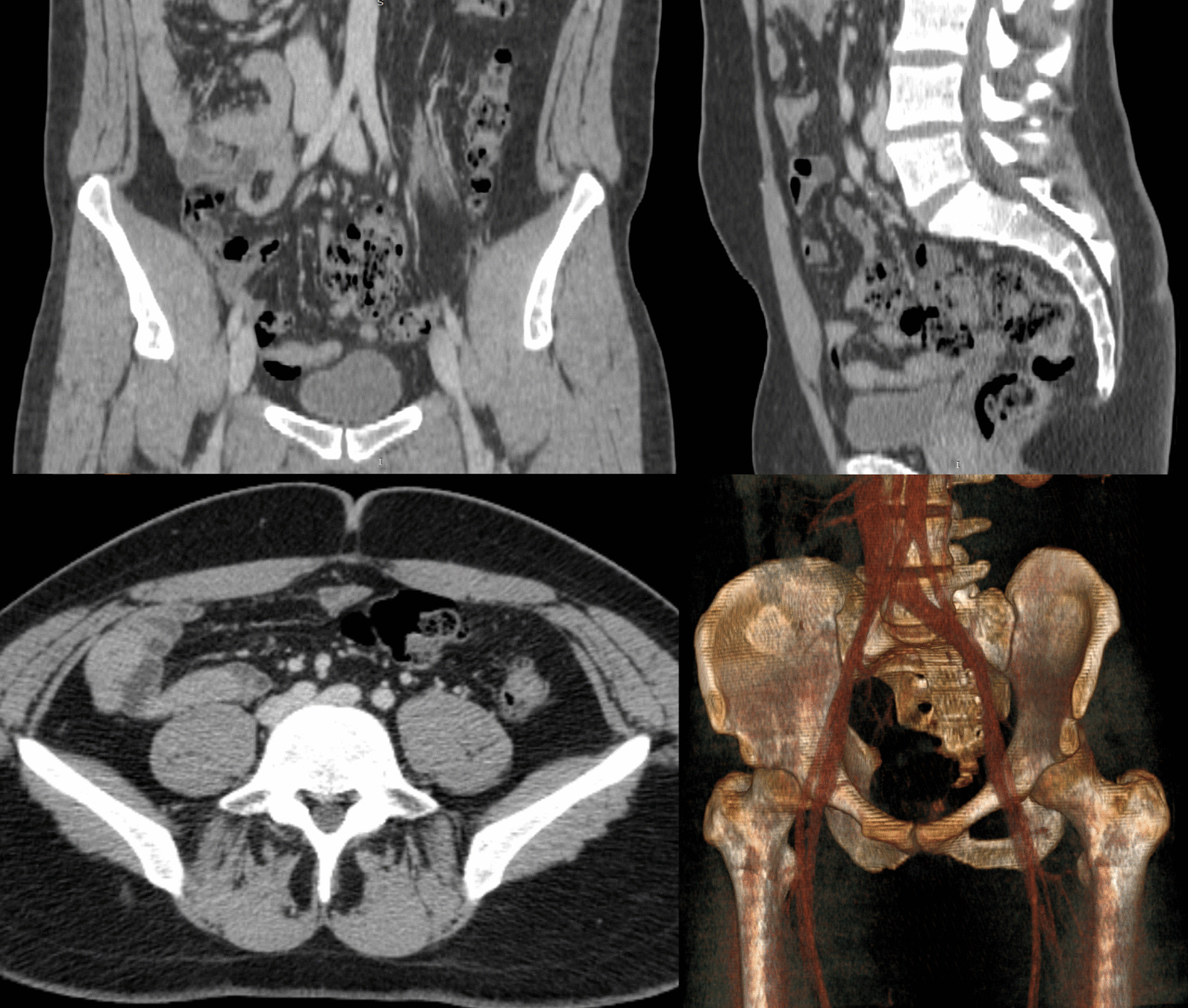
CT scans in all planes with 3D rendering demonstrate severe compression of the left common iliac vein by the right common iliac artery. 3D rendering demonstrates MTS-associated changes of the vein underlying the artery

**Fig. 3:**
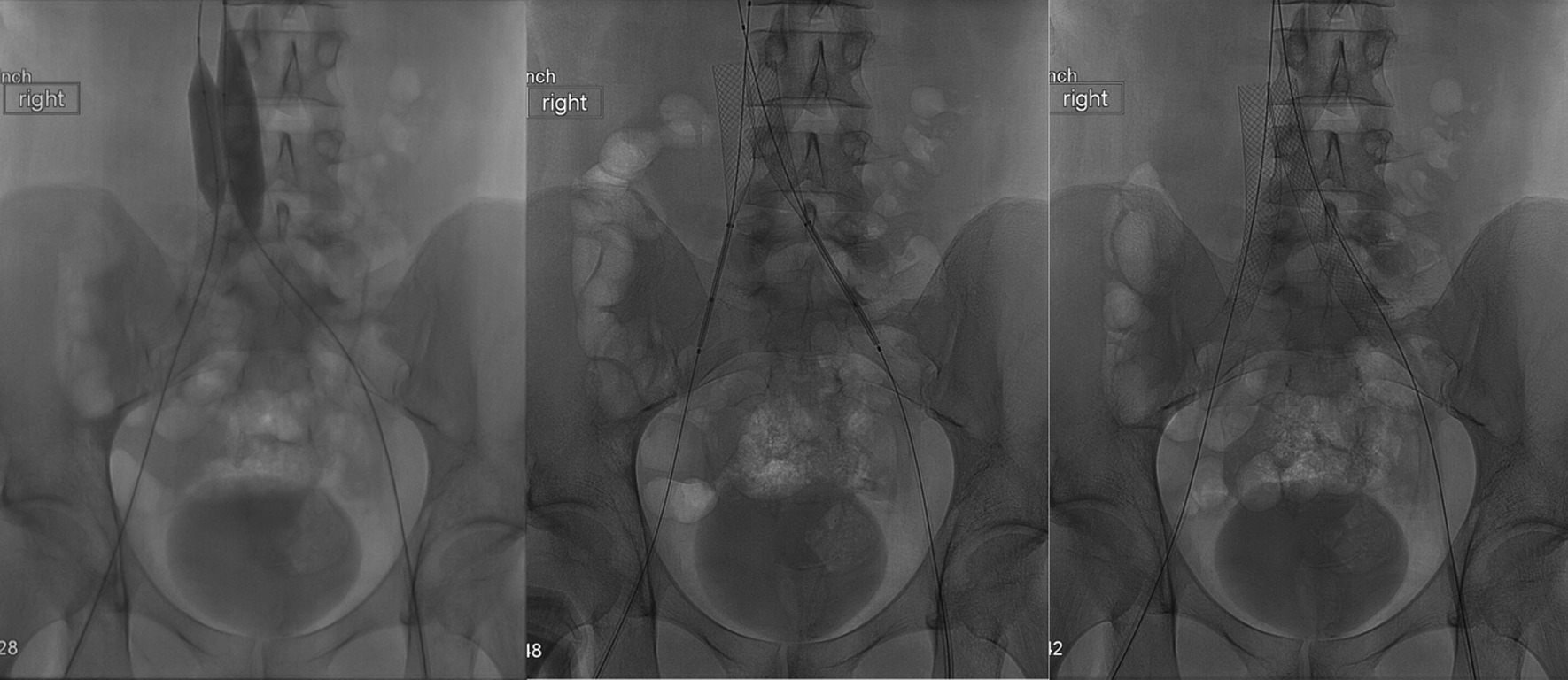
Intraoperative venograms show ballooning (left), stent deployment (middle), and completion of deployment (right). Stents were placed bilaterally after Doppler flow analysis showed diminished blood flow in both left and right internal iliac veins

**Fig. 4:**
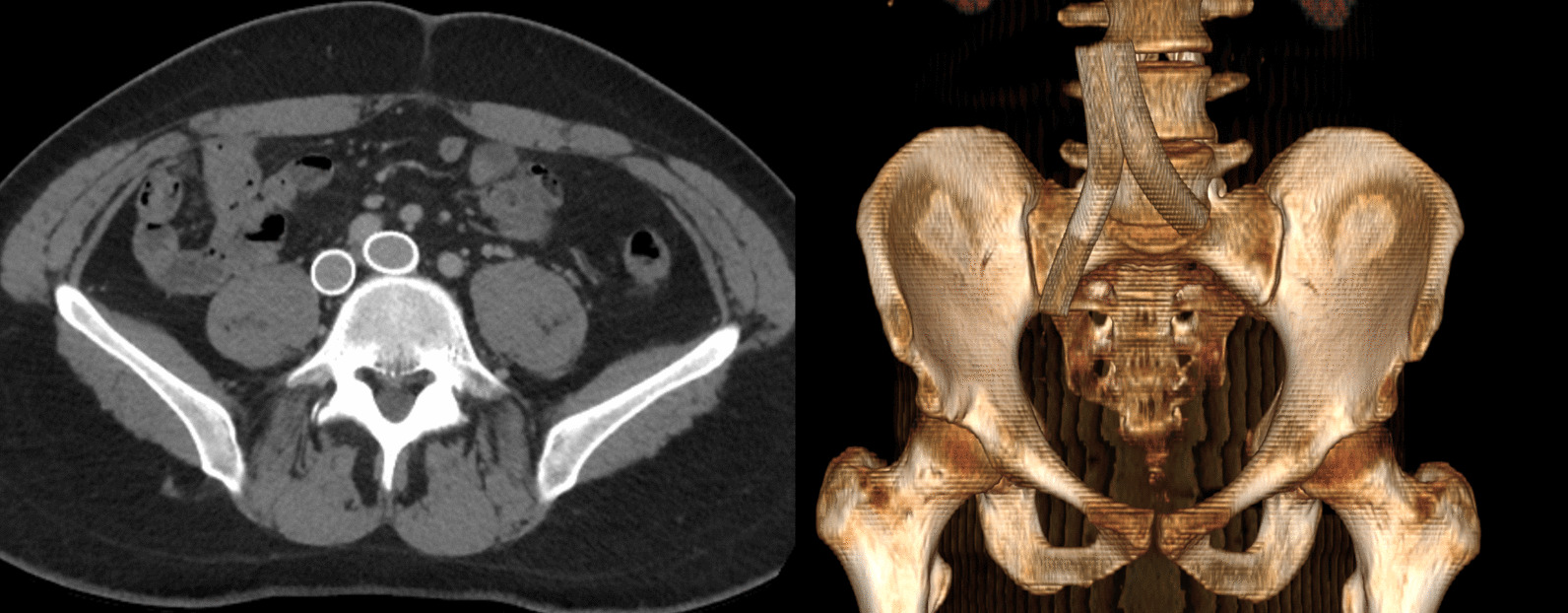
Postprocedure CT with 3D rendering demonstrates placement position of bilateral common iliac vein stents with patent luminary blood flow through the stents

At this time, the differential diagnosis was still broad. Trousseau’s syndrome was on the differential but was ruled out. Oncologic work-up was negative for malignancy. Laboratory values were within normal limits. The patient had not experienced another seizure. Work-up included full-body CT scan, which revealed MTS (Fig. 2). Hematological profile was also within normal limits, and MTS was postulated to be the cause of the patient’s clotting and was added to the differential for the underlying cause of his symptom profile. Treatment options for MTS were discussed including long-term anticoagulation therapy as a minimally invasive option and venoplasty as an invasive option. The patient wished to proceed with the more definitive option and elected to undergo endovascular venoplasty with stent placement. In March 2019, Interventional Radiology placed bilateral common iliac and right external iliac venous stents to attempt definitive treatment. Stents were placed bilaterally after Doppler flow analysis showed diminished blood flow in both left and right internal iliac veins. Post-catheterization CT with three-dimensional (3D) rendering showed placement position of and patent luminary blood flow through the stents (Figs. 3, 4).

Postoperatively, the patient was referred to hematology for follow-up care. It was deemed safe to discontinue heparin and switch to daily aspirin 4 weeks after venoplasty. Six days after discontinuation of heparin, the patient experienced another thrombotic event presenting with left leg pain and shortness of breath. Diagnostic workup, including CT, was significant for PE, and the patient was taken to the interventional radiology suite.

**Fig. 5:**
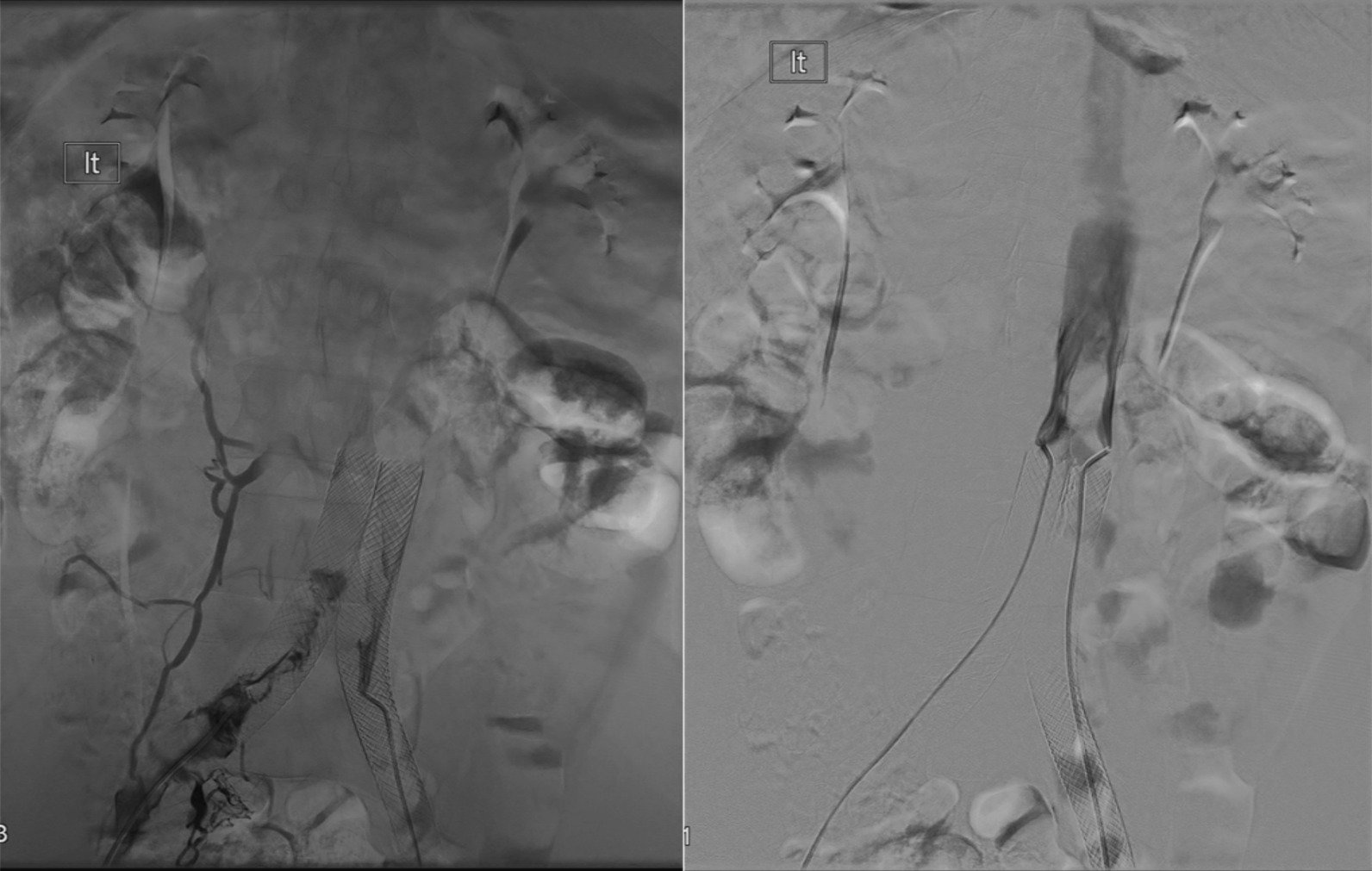
Venography 5 weeks post-stent placement shows stents in place complicated by severe intrastent and intravenous thrombosis extending into the inferior vena cava

The right popliteal vein was accessed in an integrated fashion using a six French vascular sheath. A venogram was performed. A guidewire and catheter were advanced into the common femoral veins bilaterally, and simultaneous iliac venograms were performed. Catheters were then advanced bilaterally into the common iliac stents, and IV contrast was injected. Significant thrombus was demonstrated within the right proximal external iliac vein and bilateral stented common iliac veins. The thrombus also extended proximally into the inferior vena cava (IVC) immediately above the stents as well as distally into the left femoral and popliteal veins (Fig. 5). Pharmacomechanical thrombectomy was then performed through each access site, from the left popliteal vein through the left iliac veins and into the IVC, and also from the right external iliac vein into the IVC.

**Fig. 6:**
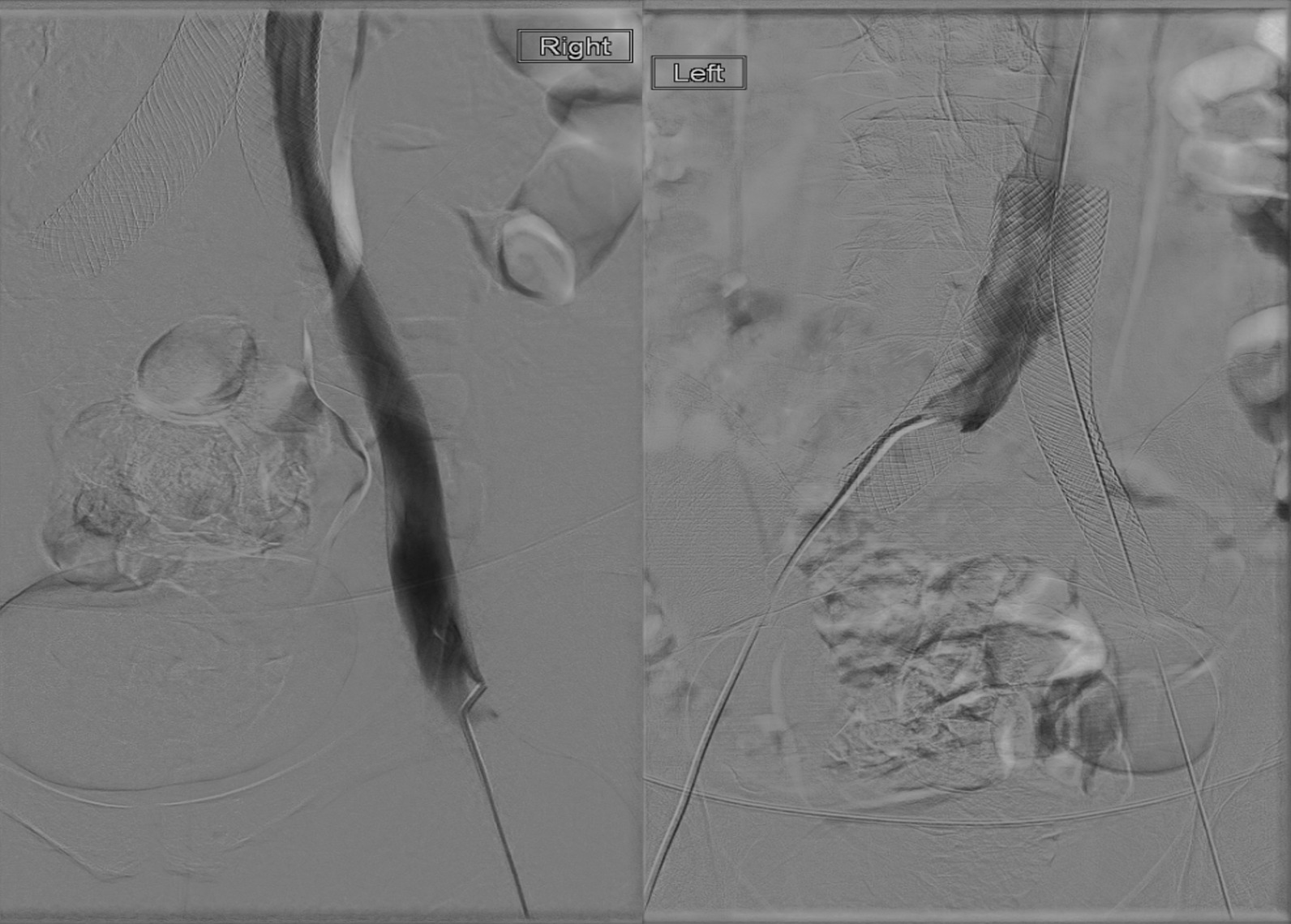
Venography shows patent flow 24 hours after inpatient pharmacomechanical thrombectomy

Following thrombectomy, 20 cm infusion length catheters were deployed bilaterally. tPA infusion was initiated through each infusion catheter. In addition, to treat the nonocclusive thrombus within the distal left femoral and popliteal veins, tPA was also infused through the access sheath at that site. All sheaths and catheters were secured to the skin, and weight-based heparin was continued. Twenty-four hours later, the patient underwent fluoroscopy which confirmed patency of the treated segments (Fig. 6). The patient remained inpatient for monitoring for the next 24 h and was discharged home on apixaban for chemoprophylaxis. The patient tolerated the procedure without complication.

The patient has since reported no additional seizures, clotting events, or emergency department visits. No other significant disorders have been found, including testing for Factor V Leiden, cancer, thyroid disorders, and patent foramen ovale. Despite the negative hematological testing, the patient remains on lifelong Coumadin in the event of an undiscovered hypercoagulopathy. The care team theorizes that the seizures resulted from hypoxia due to MTS-induced hemostasis and associated thrombotic events, the high-altitude location of his residence at the time he experienced the seizures, and shallow breathing during sleep.

## Discussion

This report of a 29-year-old male with a severe case of MTS that was treated with bilateral common iliac stent placement is unique for several reasons. The number of reports of males with MTS is few; thus, this case adds to the limited understanding of the presentation and clinical course of MTS in males. This case involves seizure activity of ambiguous etiology, distinguishing it from other MTS documentation in medical literature. Furthermore, this case provides a valuable incentive to broaden the scope of the differential diagnosis in the work-up of symptomatic patients.

### May–Thurner syndrome

MTS is characterized by extrinsic venous compression by the arterial system against bony structures in the iliocaval region. Although MTS varies in presentation, the most common is due to constriction of the left common iliac vein between the superimposed right common iliac artery and the lumbar spine. Other variations such as right-sided MTS and compression of the inferior vena cava (IVC) by the right common iliac artery can occur [2, 4, 16].

MTS is caused by a persistent pulsatile pressure from the overlying common iliac artery that aggravates the endothelium of the left iliac vein, which causes the compensatory formation of bands, colloquially known as “spurs.” These obstructions to laminar blood flow are most commonly asymptomatic unless contributing circumstances such as surgery, pregnancy, or a post-partum state trigger deep venous thrombosis [17]. The combination of tissue changes and the narrowing of the vessel causes a local procoagulation environment and distal stasis due to diminished flow, leading to thrombosis or thromboembolism.

The incidence and prevalence of MTS are unknown and are likely to be underestimated due to the occult nature of the disorder and lack of required treatment [6, 18–20]. Kibbe *et al.* [6] found that, when analyzing 50 abdominal CT scans of patients (mean age 50 years) with abdominal pain minus indicative left lower extremity symptoms, 25% had hemodynamically significant lesions (that is, > 50% stenosis) present.

### Venous spurring

A venous spur forms due to the repetitive trauma of a vein that results in an accumulation of collagen and elastin within the venous wall [21]. Negus *et al.* [22] postulated four mechanisms of venous spur formation: (1) congenital formation, (2) development of mural thrombi, (3) adhesion and formation of thrombi originating in veins in the area compressed by the artery, and (4) fibrosis directly caused by compression and pulsation by the artery.

Without regard to the original cause, venous spur formation may lead to decreased blood flow in the area, inducing varying degrees of hemostasis. Subsequent thrombi may continue to accumulate in the region, creating the potential to form a widespread thrombosis [21]. The occurrence of venous spurring specifically near the confluence of the left common iliac vein, as seen in MTS, varies. Studies analyzing the presence of venous spurs in autopsied patients varied in prevalence due to differing sample sizes, which ranged from 32 to 430, while percentages of cadavers with venous spurs ranged from 14% to 62% [21–25]. Although the mechanism of venous spur formation may differ, its formation is a foundational event that contributes to the presentation of MTS.

### Hypoxia-driven cardiogenic seizures

The causes of seizures include neurological dysfunction, vascular abnormalities, cancer, infectious processes, and drug-induced [26]. Most pertinent to the presented case is the well-documented association between pulmonary embolism and seizures [27, 28]. Although acknowledged, seizures have not been shown to be a common side effect of PE [29]. The pathophysiology suggests that PE may cause transient right ventricular failure that leads to decreased cardiac output and transitory cerebral hypoperfusion with the potential to trigger “hypoxia-driven cardiogenic seizures” [30]. Given that MTS is highly associated with an increased risk of thrombo-emboli, it is plausible that a condition causing transient hypoxia could lead to seizures.

### Endovascular treatment

As MTS is an anatomical condition, endovascular treatment to correct the mechanical obstruction coupled with anticoagulation therapy is preferable to anticoagulation alone in patients with symptomatic MTS [18, 31–33]. Endovascular balloon venoplasty with stenting is the most common approach to definitive treatment of symptomatic MTS. However, complications have been reported. One study revealed complication rates as high as 40% [34], suggesting that further research is needed to develop a standardized evidence-based approach in the management of MTS to decrease the risk of immediate and long-term difficulties. Stent thrombosis, stent stenosis, and stent migration were among the most common complications observed; one patient underwent open heart surgery for stent retrieval, so rare a complication that only one other incident has been reported [34, 35]. However, conclusions are mixed, with some studies reporting lower complication rates after iliac venous stenting [36]. One report demonstrated long-term success with stenting followed by at least 1 year of anticoagulation with oral rivaroxaban [33].

## Conclusion

Hypoxia with respiratory acidosis cannot be eliminated as the cause of our patient’s seizures, which have not recurred since stenting and prophylactic anticoagulation. Hypoxia-driven cardiogenic seizures are one possible explanation. His care team hypothesizes that the seizures resulted from a combination of hypoxia due to high altitude, shallow breathing during sleep, and MTS-induced hemostasis and associated thrombotic events. For patients with lower limb venous thrombosis who are otherwise healthy, MTS should be considered in the differential diagnosis. The epidemiology suggests that MTS is more common among females; however, reports of male patients with MTS are increasing and should be considered for a male patient presenting with unexplained DVTs. Common iliac ultrasound is difficult but can be done. CT venography can be used to confirm the diagnosis. Endovascular treatment should be followed by extended prophylactic anticoagulation therapy until the patient is determined to no longer be at risk for thrombosis.

## Data Availability

Not applicable.

## Abbreviations

MTS: May–Thurner syndrome
DVT: Deep vein thrombosis
CT: Computed tomography
ECG: Electrocardiogram
PE: Pulmonary embolism
tPA: Tissue plasminogen activator
IVC: Inferior vena cava
PTE: Pulmonary thromboembolism
